# Drastic Effects on the Microbiome of a Young Rower Engaged in High-Endurance Exercise After a Month Usage of a Dietary Fiber Supplement

**DOI:** 10.3389/fnut.2021.654008

**Published:** 2021-04-30

**Authors:** Mariliis Jaago, Uku Siim Timmusk, Tõnis Timmusk, Kaia Palm

**Affiliations:** ^1^Protobios Llc, Tallinn, Estonia; ^2^Department of Chemistry and Biotechnology, Tallinn University of Technology, Tallinn, Estonia

**Keywords:** microbiome, endurance sports, rowing, dietary fiber, athlete, junior, case study

## Abstract

Food supplements are increasingly used worldwide. However, research on the efficacy of such supplements on athlete's well-being and optimal sports performance is very limited. This study performed in junior academic rowing explores the effects of nutritional supplements to aid to the high energy requirements at periods of intense exercise. Herein, the effects of prebiotic fibers on the intestinal microbiome composition of an 18-year-old athlete exercising at high loads during an 8-month period in a “real-life” setting were examined using next-generation sequencing analysis. Results demonstrated that although the alpha diversity of the subject's microbiome drastically decreased [from 2.11 precompetition to 1.67 (*p* < 0.05)] upon fiber consumption, the *Firmicutes*/*Bacteroidetes* ratio increased significantly [from 3.11 to 4.55, as compared with population average (*p* < 0.05)]. Underlying these macrolevel microbial alterations were demonstrable shifts from acetate- to butyrate-producing bacteria, although with stable effects on the *Veillonella* species. To our knowledge, this a unique study that shows pronounced changes in the gut microbiome of the young athlete at the competition season and their favorable compensation by the dietary fiber intake. The data here expand the overall understanding of how the high energy needs in high-intensity sports like academic rowing could be supported by dietary fiber supplement consumption.

## Introduction

The microbiome contributes to thehomeostatic regulation of different tissues in our body (1) with the largest and most diverse cluster of microorganisms inhabiting the gut (2). These core functions are linked to the production of essential and extremely diverse metabolites such as vitamins (vitamin B_12_, folic acid, or vitamin K), bile acids, neurotransmitters (serotonin, dopamine, acetylcholine), and short-chain fatty acids (SCFAs: acetic acid, propionic acid, and butyric acid) (3). Diet and the level of physical activity are the main determinates for altering the gut microbiota (4). Increases in bacterial diversity and a proliferation of taxa responsible for the production of SCFAs, such as butyrate, are among the most pervasively observed microbial alterations with exercise (5).

Athlete microbiomes have been found to contain distinct microbial compositions defined by elevated abundance of *Veillonellaceae, Bacteroides, Prevotella, Methanobrevibacter*, or *Akkermansia* (6). Cardiorespiratory fitness in exercising subjects was associated with higher abundance of butyrate-producing bacteria by the *Clostridiales, Erysipelotrichaceae, Lachnospiraceae*, and *Roseburia* families (7). Exercise type along with athlete diet patterns (bodybuilders: high protein, high fat, low carbohydrate, and low dietary fiber diet; distance runners: low carbohydrate and low dietary fiber diet) was significantly associated with the relative differential abundance of *Faecalibacterium, Sutterella, Clostridium, Haemophilus, Eisenbergiella, Bifidobacterium*, and *Parasutterella* in bodybuilders and distance runners (8). Variation in genera was suggested to be linked to the variance in species' composition across different types of sports (9). So, athletes participating in sports with high dynamic and static component like academic rowing displayed greater abundance of *Bacteroides caccae* (9). The effects of exercise on gut microbial microorganisms were concluded to depend significantly on its intensity and timing with the notion that the microbiota could also influence muscle mass, as reported by Ticinesi et al. (10). Excessive exercise among professional athletes disturbs the homeostasis of the gut microbiota [reviewed in (7)]. Physical exertion at a very high level for a prolonged time means that the whole body initiates a defense response because of oxidative stress, intestinal permeability, muscle damage, systemic inflammation, and immune responses (11). It has been observed that endurance athletes present a high prevalence of upper respiratory tract infections and gastrointestinal troubles, including a “leaky gut,” disruption of mucous thickness, and higher rates of bacterial translocation (12, 13). Overall, all these studies suggest that the gut microbiome affects exercise performance and vice versa.

Diet has a major impact on gut microbiota composition, diversity, and richness. Dietary supplements that employ non-digestible dietary fibers have been developed for several decades as prebiotics to support growth of beneficial GI microbiota (14). Dietary fibers can be found in plants, bacteria, and fungi and can be chemically synthesized (15). The health effects of these dietary fibers have extensively been reviewed and accepted worldwide (14). It has been concluded that the extent by which different fiber types are utilized or fermented by the GI microbiota is structure dependent and relies on the metabolic capabilities of the individual's microbiome (16). Virtually, all fibers induce specific shifts in microbiota composition due to competitive interactions; however, which of these shifts contribute to health, or if at all, is not known (17, 18). The commensal bacteria ferment non-digestible fiber primarily into CO_2_, H_2_, and CH_4_ and SCFAs (19). Most of the SCFAs produced in the intestine are absorbed by the host to contribute to energy and beneficial metabolites (20) that are also used as carbon and energy sources by other specialized bacteria including reductive acetogens, sulfate-reducing bacteria, and methanogens (21).

Multiple lines of evidence support the hypothesis that modification of the microbial community through diet could be an effective tool to improve athlete's health (14) performance and energy availability while controlling redox levels and inflammation (22). Endurance diets are rich in protein (1.2–1.6 g/kg/day), which produce a range of potentially harmful compounds in the intestine in addition to SCFA productions (22). There are only a few demonstrated studies in athletes consuming prebiotics (23). Research has suggested the validity of probiotics to improve training parameters and increase training capabilities (24).

Nutritional supplements are popular among athletes to improve performance and physical recovery. Long-term (10 weeks) protein supplement (whey isolate and beef hydrolysate) consumption by cross-country runners, however, decreased the presence of health-related taxa including *Roseburia, Blautia*, and *Bifidobacterium longum* and increased the abundance of the Bacteroidetes phylum (25). However, it appeared that protein overconsumption was an offset by a higher intake of indigestible polysaccharides (26).

Athletes with very high training and competition loads can have serious problems getting the necessary amount of energy from regular food. Clark and Mach (2016) reported that diets recommended for athletes likely influence gut microbiota by reducing diversity because these diets include insufficient dietary fiber (27). In addition, a recent study showed that *Veillonella atypica* has a beneficial impact on the performance of elite athletes (6). Based on these findings, we decided to look for dietary ways to increase beneficial bacteria for better athletic performance and faster recovery, in particular considering that nutritional supplements are popular among athletes. We hypothesized that microbial profiles of the young rower might share features of those previously described in endurance sports studies but that this might change in response to the dietary changes upon dietary fiber supplement intake.

## Materials and Methods

### Case Presentation

At the time of the study, the male athlete was 18 years old and was studied over the course of a 31-week period during the 2019 race and training season preceding the world championship competition in U19 category of academic rowing. By this time, the subject had been undertaking rowing for 4 years and had not previously sought any conditioning or dietary advice. Furthermore, the athlete was not on any prescribed medication and was a non-smoker, and his usual diet was previously supplemented with whey protein [SiS (Science in Sport) Limited products] only. In the 7 months prior to the supplement intake, the subject held a normal diet that was alike on a daily basis, comprising mostly of meals that were high in carbohydrate and protein, medium in fat, and modest in dietary fiber. The athlete was fully informed of the study aims and confirmed participation in the study by signing a consent form, understanding that the parameters of health were not associated with the study and that the subject was not physically harmed by the study. This study was approved by Protobios (1-05/2019).

### Goals of the Study

The primary goals of the support provided were to (a) get insights into the subject's intestinal microbial community during periods of high exercise and (b) examine the effects of dietary fiber intake to the bacterial compositions associated with energy production.

### Diet and Activity Recordings and Microbiome Sampling

**Figure 1:**
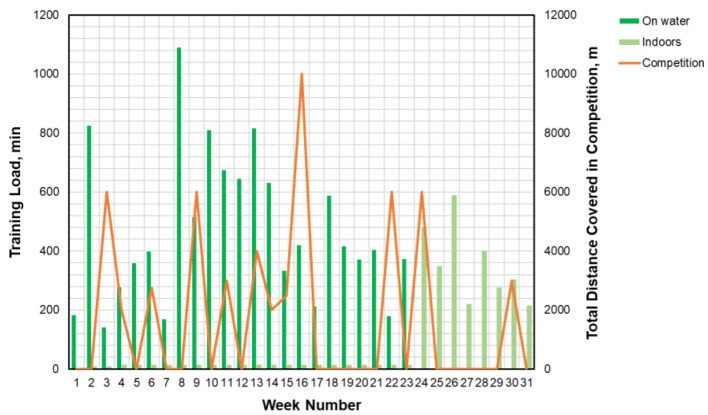
Training program undertaken throughout the study period. Electronic exercise diary (www.sportlyzer.com) was kept for water and ergometer training hours throughout the study period. On the left *y*-axis—training load in minutes per week, min/week (Rowing on the *Water* and *Indoors*); on the right *y*-axis—distance in meters per week, m/week (*Competition/m*); on the *x*-axis—duration of the study period in weeks. Samples for microbiome analysis were taken at weeks 1, 27, and 31. *On water*, training on the water; *Indoors*, training on rowing ergometer; *Competition*, competition calendar of the rower.

The participant was informed to maintain the usual dietary habits throughout the study. Body composition estimates were made preseason (sample #1) and post study (sample #3) with no substantial change (BMI 23.3 ± 0.2, fat percentage of 8.4 ± 0.0) according to the medical sports health survey records. Based on diet recall, the usual macronutrient intake was assessed using image-based dietary assessment software (NutriData, National Institute for Health Development, Estonia). We used this tool to ascertain the usual eating patterns of the subject including type, frequency, and amount of foods consumed. Foods consumed were matched to the nutritional analysis for the specific menu items that had been coded in NutriData. If not consumed from the menu, the item was coded against the most appropriate matching food. On average, across the study period, the athlete consumed 2,560 ± 750 kcal/day, and the estimated macronutrient intake was 23 ± 7.12% protein, 52 ± 19.1% carbohydrate, 26 ± 19.3% fat, and 15 ± 4.5 g fiber. The consumption of the nutrient supplement (*Food, not only for thought*, Elsavie, Estonia) started on week 27 and continued on a daily basis until week 31 (altogether 30 days), with intake immediately after breakfast, and the recommended daily intake [1.5 tablespoons (1.5 tbsp/20 g) mixed with water] was not exceeded. The dietary supplement provided to the participant as the prebiotic mix (20 g) included dietary fiber (8.79 g), consisting of resistant starch (2.25 g), arabinoxylan (2.05 g), citrus fiber (2 g), beta-glucans (1.03 g), inulin (1.03 g), and rye fiber (0.57 g). The athlete kept the training diary in Sportlyzer (Sportlyzer, Estonia). The 31-week high-training and intensive competition program is presented in Figure 1. The mean physical activity (8 months duration) of the subject during the study was 472 min/week of water rowing, 354 min/week of indoor rowing, and 60 min/week of stretching exercises (Figure 1). Since the time the first microbial sample (#1) was taken, the subject was participating in a series of national and international competition activities including five international and 11 local competitions (53.25 km total of total race distance) following the planned program as in Figure 1. The second sample (#2) was taken at week 27, by the end of the water season and at early weeks of the indoor rowing season. Thereafter, the subject began to take the dietary fiber supplement as recommended for 30 days. A third sample (#3) was taken at week 31. The seasonality of sampling was spring (#1), autumn (#2), and winter (#3), respectively.

### Microbiome Assessment

Microbiome composition was determined on three occasions (week 1, week 27, and week 31, Figure 1). Samples were self-collected from morning stool samples by using the commercially available kit (INTEST.pro, BIOMES NGS GmbH, Germany) in accordance with the specifications laid out by the manufacturer. The first two samples were taken at normal nutrition (at the start of and after the active competition period), followed by 1 month of dietary supplement intake to investigate the dynamics of the intestinal microbiome and the effects of the fiber supplement on the microbiota. Collected samples were transported to the lab according to the service provider's instructions where the microbiome composition was analyzed *via* 16S rRNA gene amplification and sequencing by BIOMES NGS GmbH (Germany). In brief, microbial genomic DNA from fecal material was extracted by the bead-beating technique, the V3–V4 region of the 16S rRNA gene was amplified, and sequencing was performed on the Illumina MiSeq platform using a 2 × 300-bp paired-end protocol (Illumina, San Diego, CA, USA). These DNA sequencing techniques were then used to generate data outputs that provided a comprehensive bacterial taxonomic profile of the subject (28) in comparison with the average microbiome of the European population as the evidence indicates that microbiome may vary by geography (29).

### Data Analysis and Statistics

Different packages of MS Excel (based on MS Excel 2011) and licensed MedCalc (version 19.1.6) statistical analysis programs were used for taxonomic, functional analysis, and visualization of bacterial composition data obtained from microbiome sample study reports. Spearman's correlation coefficients *r*_s_ were calculated for comparing abundances of genera at two time points. Statistical significance of differences of Shannon's indices (α-diversity) across time points was assessed using Hutcheson's modified *t*-test with a significance level of *p* < 0.05. Statistical significance of differences between the athlete's characteristics and the control group (general population) was assessed using the single mean *t*-test with a significance level of *p* < 0.05. Relative abundance values of bacteria on genus and species level at different time points were compared using non-parametric Wilcoxon rank sum tests (also named the Mann–Whitney *U*-test or Mann–Whitney–Wilcoxon test) with a significance level of *p* < 0.05.

## Results

### General Diversity, Phylogenic Composition, and Core Gut Microbiota of the Athlete

**Figure 2:**
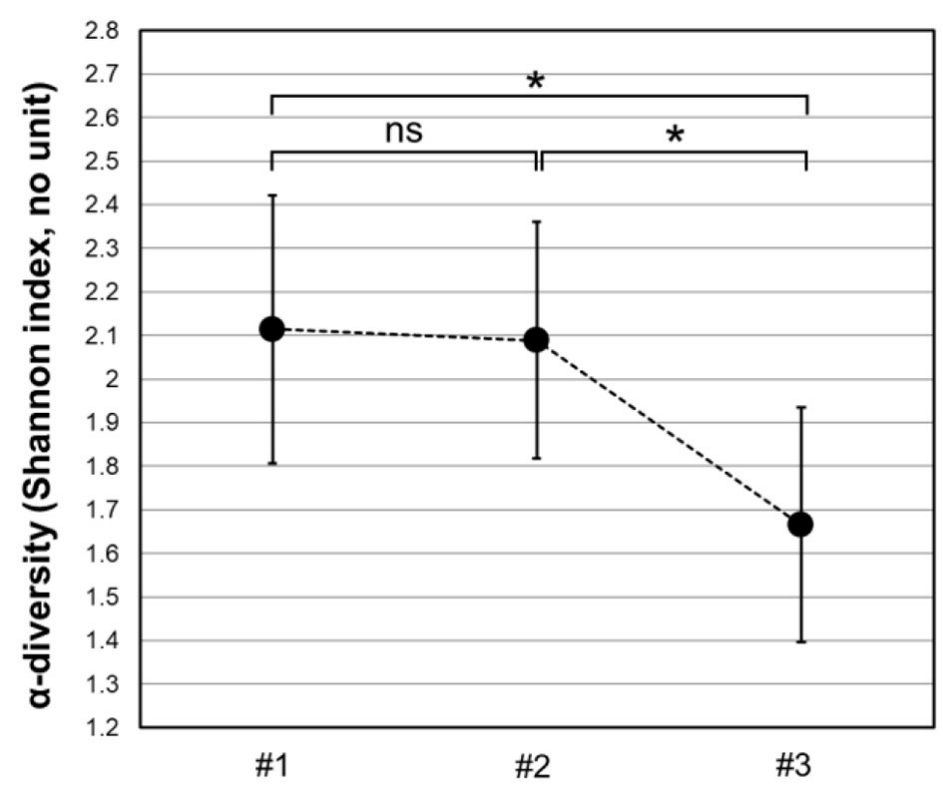
Changes in the microbiome diversity over time and drastic effects on its relative diversity within bacterial families upon the dietary fiber consumption. The Shannon index expressed as a measure of the incidence and frequency of bacterial families in the test samples, ranged from 2.11 (#1) to 2.08 (#2), which was not a statistically significant difference (*p*-value > 0.05, Hutcheson's modified *t*-test, denoted *ns*—not significant). One month after consumption of the fiber mixture, the Shannon index dropped to 1.67, which was a significant decrease in the family diversity values (*p*-value < 0.05, Hutcheson's modified *t*-test, denoted by *). Error bars represent 95% CI for the calculated Shannon index. *y*-axis—Shannon index, without units. *x*-axis—sampling times. #1, time point week 1 (baseline); #2, time point week 27 (high training and competition period); #3, time point week 31 (after 30 days of dietary supplement intake).

The Shannon index indicating the diversity of bacterial families present in the samples of the subject ranged from 1.67 to 2.11 (Figure 2). At the beginning and end of the training period, the Shannon indices were similar (2.11 and 2.08, respectively), concluding that the microbiome diversity did not change significantly at times of high competition (Hutcheson's modified *t*-test, *p*-value > 0.05). After the dietary fiber mix intake, the Shannon index dropped to 1.67 with a significant decrease in community diversity (Hutcheson's modified *t*-test, *p*-value < 0.05). The Shannon index of the control group was 1.63 (data not shown).

The evenness of the distribution of species in communities showed that at high exercise, microbiome uniformity indices were similar (J1 = 0.54 vs. J2 = 0.53), whereas, after the fiber intake, *J* showed a substantial drop (to J3 = 0.42). Although, also the evenness values of the microbiota were the lowest after the fiber mix diet (changed from 0.53 to 0.42), the statistical significance of the reduction could not be concluded. The uniformity index for the control group was *J* = 0.41. These data allowed us to conclude that at the family level, the athlete's microbiome at high training and competition loads (samples #1 and #2) were more diverse and more balanced (even) than after the dietary fiber consumption (sample #3). Intense exercise accompanied by a high intake of dietary fiber did not lead to the increased diversity of gut microbiota as was initially expected.

**Figure 3:**
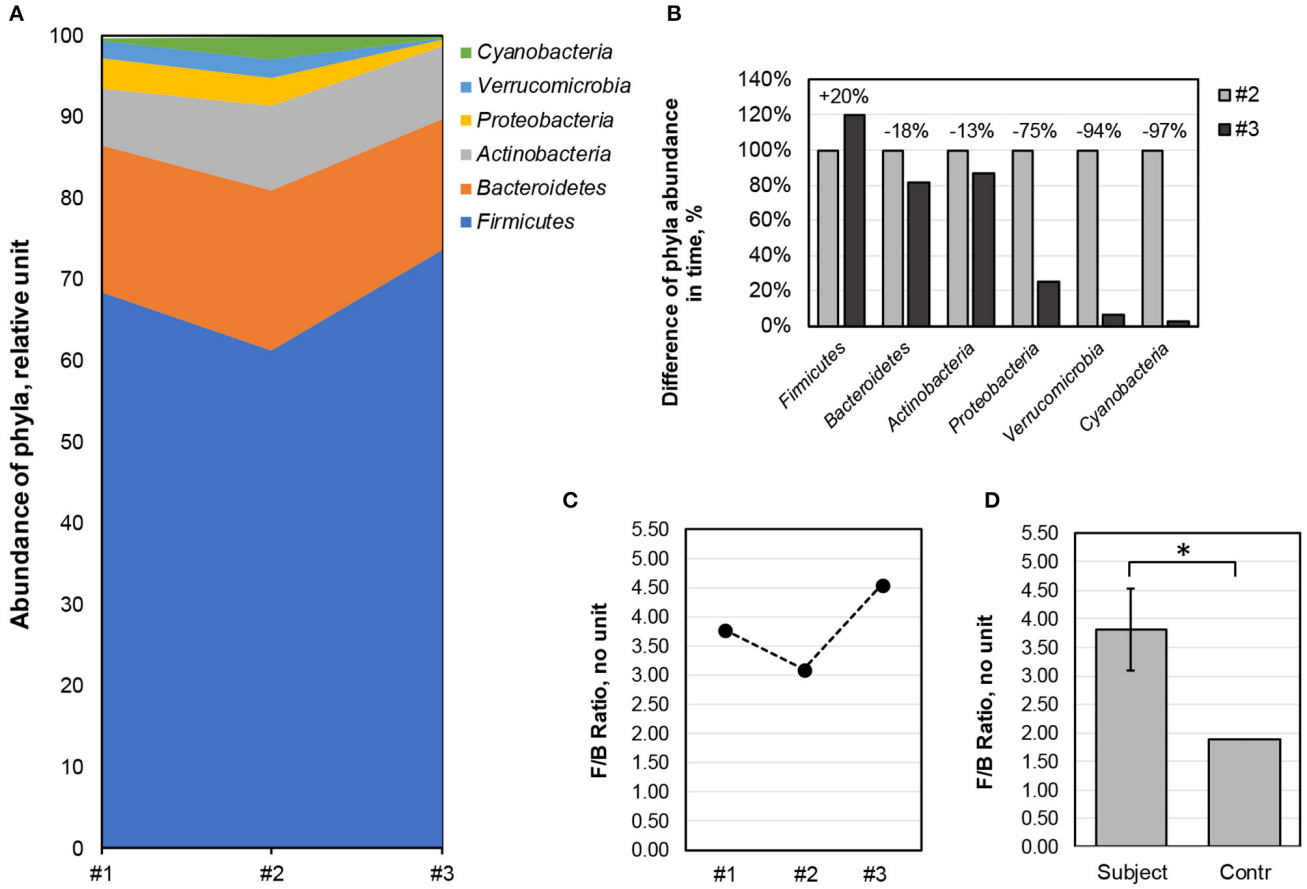
Dynamic changes in bacterial phyla composition over the study period. **(A)** Changes in the abundance of the six most numerous phyla (*Firmicutes, Bacteroidetes, Actinobacteria, Proteobacteria, Verrucomicrobia*, and *Cyanobacteria*) in three serial samples (*x*-axis). *y*-axis—abundance of phyla, in relative units. **(B)** Proportional changes in the abundance of the six major phyla upon fiber consumption. The abundance of each phylum in sample #2 was equated to 100%, and the change in sample #3 relative to sample #2 is indicated above the columns. *y*-axis—relative abundance (%) of phylum in samples #2 and #3; *x*-axis—phyla. **(C)** Changes in *F*/*B* ratio over the 31-week study period, measured at three serial time points (*x*-axis). *y*-axis—*F*/*B* ratio. **(D)** The young athlete showed a high *F*/*B* ratio as compared with the control group (single mean *t*-test, *p*-value < 0.05, denoted by *). The bar graph represents the arithmetic mean of the *F*/*B* ratio in study samples and the error bars represent the standard deviation. *y*-axis—*F*/*B* ratio. #1, time point week 1 (baseline); #2, time point week 27 (high training and competition period); #3, time point week 31 (after 30 days of dietary supplement intake).

As for the phylogenic composition, a total of nine phyla were present in all the samples with the dominance of *Firmicutes, Bacteroidetes, Actinobacteria*, and *Proteobacteria* (Figure 3). The *Firmicutes* [67.8 ± 6.2 (mean abundance across three time points ± SD)] outranked *Bacteroidetes* (18.0 ± 1.8), *Actinobacteria* (8.8 ± 1.7), *Proteobacteria* (2.7 ± 1.6), *Verrucomicrobia* (1.6 ± 1.2), and *Cyanobacteria* (1.0 ± 1.4) phyla. Dietary fiber consumption had a positive effect on the abundance of *Firmicutes* (+20%), whereas, it showed drastic negative effects on *Verrucomicrobia* and *Cyanobacteria*, whose drop in abundance was almost 100%, and on *Proteobacteria, Bacteroidetes*, and *Actinobacteria* that declined by 75, 18, and 13%, respectively (Figure 3B). Analysis of *Firmicutes*/*Bacteroidetes* (*F*/*B*) ratio values showed relative stability during the study, where during the intense competition period, the reduction in *Firmicutes* [changing the *F*/*B* ratio by 17.9% (from 3.78 to 3.11)] was rescued upon fiber consumption by increases in abundance by 20% and resulting in the *F*/*B* ratio of 4.55 (Figure 3C). Compared with the control group, the *F*/*B* ratio of the athlete was significantly higher in all time points (*t*-test of one mean, *p*-value < 0.05, Figure 3D). Altogether, these results showed that dietary fiber along with high exercise loads affected the phylogenic composition by the microbiome of the athlete becoming relatively poorer at the phylum level. Overall, these results were in good agreement with data showing that training promoted relative increases in *Firmicutes* (30, 31) and that *F*/*B* ratio correlated significantly with cardiorespiratory fitness (32).

### Opposite Dynamics of Butyrate-Producing Bacteria in Periods of Competition and Upon Dietary Fiber Intake

**Figure 4:**
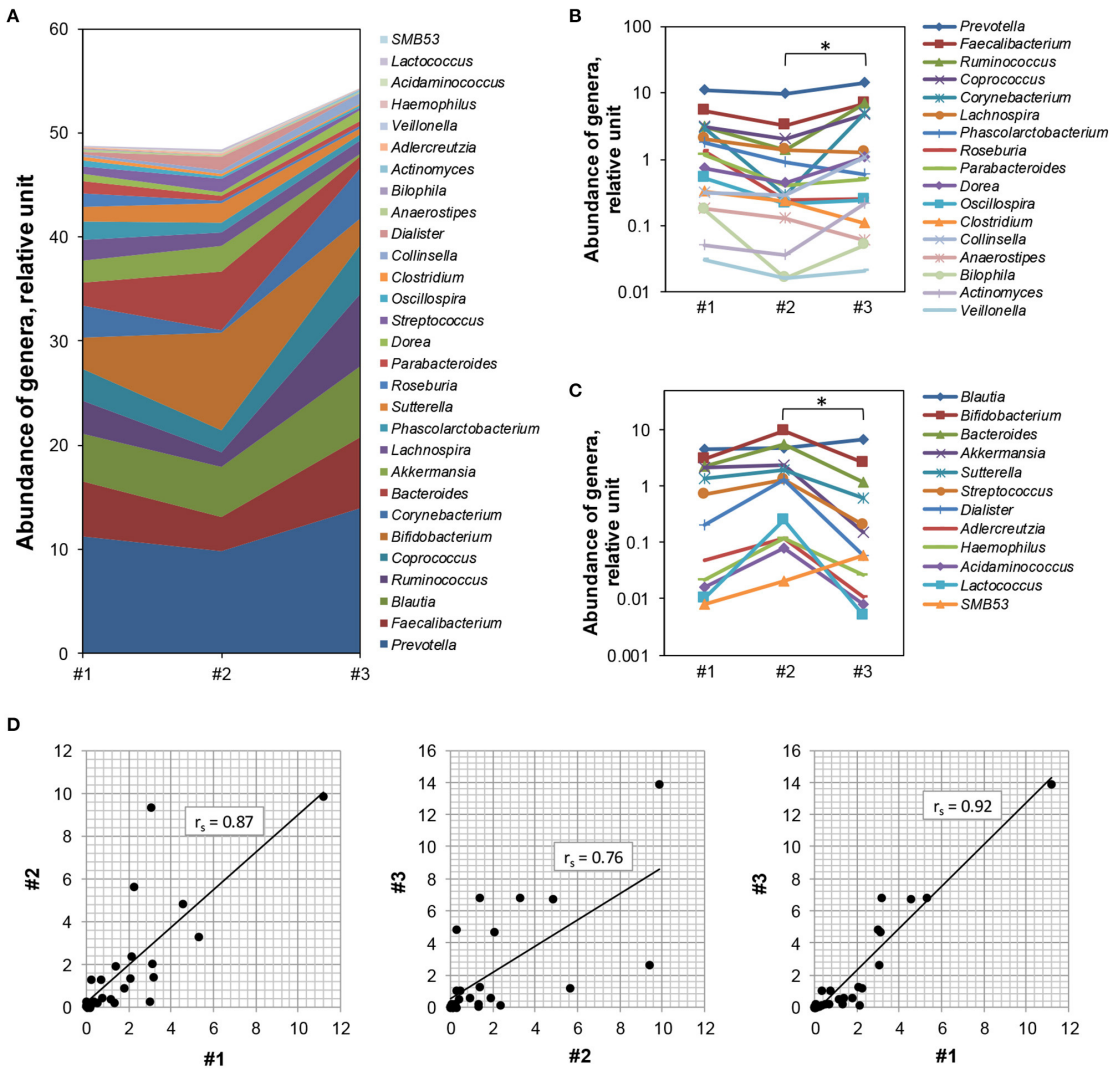
Dynamic changes in bacterial genera composition during the study period. **(A)** The bacterial genera (*n* = 29) detected in all three samples at different time points (*x*-axis) are presented. *y*-axis—abundance of genera, in relative units. **(B,C)** Those species (*n* = 17) predominantly producing acetate had higher abundance in sample #2 compared with sample #1 but were significantly lower in sample #3 (Wilcoxon rank sum test, *p*-value < 0.05, denoted by *) **(B)**, whereas, butyrate-producing species were higher in sample #3 and low in samples #1 and #2 (Wilcoxon rank sum test, *p*-value < 0.05, denoted by *) **(C)**. *x*-axis—serial time points; *y*-axis—abundance of genera, in relative units. The number of bacterial genera is plotted in logarithmic scale. **(D)** Correlations between the differential abundance of large bacterial genera (*n* = 29) in the microbiome composition. The difference was high before vs. after the intake of fiber [sample #2 vs. #3, middle graph, Spearman's correlation coefficient *r*_s_ = 0.76 (*p* < 0.0001)], as compared with that at the beginning and the end of the intense training period [sample #1 vs. #2, left graph, Spearman's correlation coefficient *r*_s_ = 0.87 (*p* < 0.0001)], and also to that at the beginning and the end of the study [samples #1 and #3, Spearman's correlation coefficient *r*_s_ = 0.92 (*p* < 0.0001)]. *x*- and *y*-axes—abundance of genera, in relative units. #1, time point week 1 (baseline); #2, time point week 27 (high training and competition period); #3, time point week 31 (after 30 days of dietary supplement intake).

The relative abundance of 77 genera, of which 29 were shared across all samples, was significantly different between samples (*p*-values < 0.05, Wilcoxon rank sum test, Figure 4). At the genus level, *Prevotella* [11.7 ± 2.1 (mean abundance across three time points ± SD)], *Faecalibacter* (5.1 ± 1.8), *Blautia* (5.4 ± 1.2), *Ruminococcus* (3.8 ± 2.8), and *Bifidobacterium* (5.0 ± 3.8) were the most abundant genera (Figure 4). The predominance of *Prevotella* compared with the families of *Bacteroides* and *Ruminococcus* indicated that the subject had *Prevotella*-predominant enterotype, e.g., enterotype II (Figure 4A). *Prevotella*'s abundance was associated with long-term fiber intake (33).Similar trends were noted also in the current study whereupon dietary fiber intake resulted in enhanced abundance of *Prevotella* that became 41.7% more abundant as compared with the previous time point (Figure 4A). It is known that extreme dietary changes can lead to wide-ranging shifts in the gut bacterial community (34). Herein, the relative abundance of the acetate-producing bacteria (e.g., *Blautia, Bifidobacterium, Sutterella* groups) and the lactate-producing bacteria (e.g., *Bifidobacterium, Streptococcus, Lactococcus* groups) increased during high training and competition period (sample #1 vs. #2), but showed decreasing patterns (except *Blautia*) by the end of the dietary supplement intake period when acetate- and lactate-consuming and butyrate-producing genera including *Faecalibacterium* increased significantly (sample #2 vs. #3, *p*-value < 0.05, Wilcoxon rank sum test, Figures 4B,C). Interestingly, the propionate-producing genera (35) showed differential patterns. *Bacteroides* and *Acidaminococcus* showed increasing trends in contrast to *Phascolarctobacterium* and *Veillonella* that decreased upon competition, with only the levels of *Veillonella* slightly rescued upon fiber consumption (Figures 4B,C).

The abundance of the shared largest 29 genera was better correlated in samples #1 and #2 (Spearman's correlation coefficient *r*_s_ = 0.87) as compared with that in samples #2 and #3 (rs = 0.76, Figure 4D). However, the strongest correlation in abundance of these major genera was observed between baseline and endpoint (samples #1 and #3, *r*_s_ = 0.92). This result was somewhat surprising. Similar to other studies (36), we also noted strong patterns of individuality of the response to exercise and diet, with a possible explanation that these activities supported the original (primary, “keystone”) bacterial community dynamics of the subject.

Taken together, high exercise along with dietary fiber intake resulted in dynamic shifts in genera composition especially in the balance of lactate- and acetate/butyrate-producing bacteria.

### Selective Effects of the Dietary Fiber Supplement on Individual Species of the Gut Microbiota

**Figure 5:**
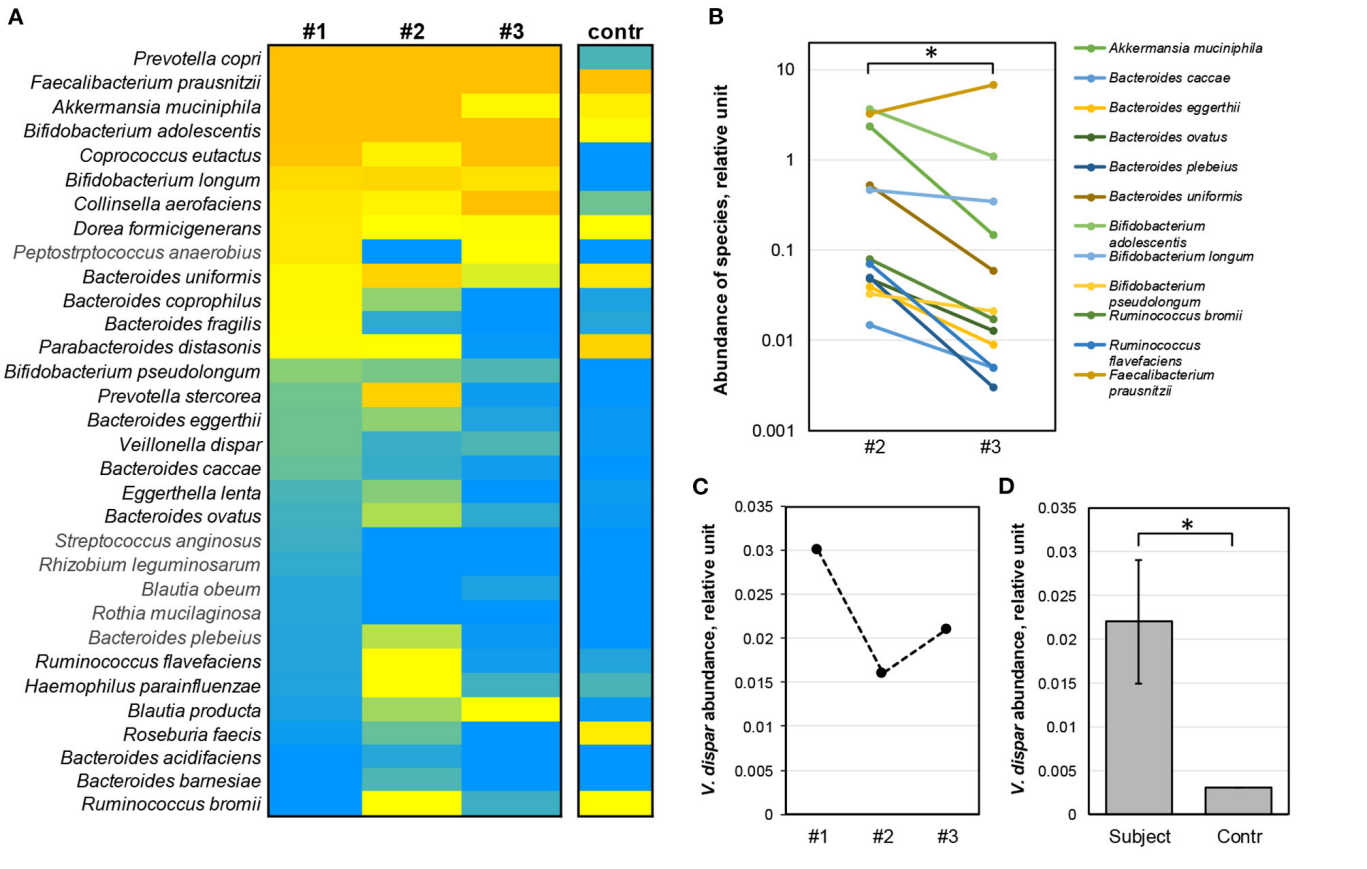
Changes in composition of the 32 most abundant bacterial species during the study period. **(A)** Dynamic changes in the abundance of observed species in different samples. Relative abundance of specific species (*rows*) in samples (*columns*) are shown in color scale with orange depicting abundance values >1 RUs, yellow >0.07, green >0.03, and blue = 0. **(B)** Abundance of species with a protective role on the intestinal mucosa (*n* = 12) showed a significant reduction after monthly fiber intake (sample #2 vs. sample #3, Wilcoxon rank sum test, *p*-value < 0.05, denoted by *); *y*-axis—number of bacterial species, in relative units. **(C)** Differential changes in the level of abundance of *Veillonella dispar* (in relative units) before the competition season (#1), after the end of the intensive competition period (#2) and after the fiber intake (#3). *x*-axis—serial time points; *y*-axis—abundance, in RU. **(D)** High abundance of *V. dispar* in the microbiome under study (*Subject*) was observed as compared with the control group (*p*-value < 0.05, single *t*-test, denoted by *). Bar graph expressed as arithmetic mean of abundance of *V. dispar* in samples and error bars depicted by standard deviation. *y*-axis—abundance of *V. dispar*, in RU. #1, time point week 1 (baseline); #2, time point week 27 (high training and competition period); #3, time point week 31 (after 30 days of dietary supplement intake).

Next, we compared the mean relative abundance of 32 individual species to identify those that were the most affected by the dietary switch. Among the studied species, the six most abundant bacterial species (*Prevotella copri, Faecalibacterium prausnitzii, Akkermansia muciniphila, Bifidobacterium adolescentis, Coprococcus eutactus, Collinsella aerofaciens*) accounted for 92.5% on average of the abundance of the top species. For most of these analyzed species, a notable variation was associated both with the intense exercise loads and the dietary fiber consumption (Figure 5). Interestingly, *A. muciniphila* (*Verrucomicrobia*) that produces both propionate and acetate (37, 38) showed decreased proportions upon fiber consumption and was replaced by the abundance of the butyrate producer *C. aerofaciens* (Figure 5A). Another major shift upon fiber consumption was noted in the abundance of *C. eutactus* with known beneficial effects on butyrate production (39). However, the abundance of 12 species attributed with a protective role on the intestinal mucosa reduced significantly after dietary fiber intake (*p*-value < 0.05, Wilcoxon rank sum test), from on average 50.4 to 33.5% of abundance of the detected species (Figure 5B). Herein, two of the five species which were among the most significantly affected were the *Bacteroidetes* spp. Also, the species of *Akkermansia, Bacteroides, Bifidobacterium*, and *Ruminococcus* with protective functions on the intestinal mucosa showed a significant decrease upon fiber intake, whereas, *F. prausnitzii*, one of the major manufacturers of butyrate, showed increased abundance upon dietary fiber (Figure 5B). *Veillonella dispar* species were specifically monitored because of their potential impact on performance enhancement as a lactic acid-utilizing community (6). Although, changes in *V. dispar* abundance during periods of high exercise load and dietary fiber intake were detected, the statistical significance of these findings could not be determined (Figure 5C). It is noteworthy that the number of *V. dispar* in the microbiome of the young athlete was significantly higher than of the control group (Figures 5C,D, single mean *t*-test, *p*-value < 0.05). The observed changes in the abundance of bacteria producing SCFAs associated with energy consumption of the skeletal muscle (40) supported the initial work hypothesis that dietary fiber intake could facilitate athletic endurance by favorable shifts in microbial composition.

## Discussion

This work investigated the effects of dietary fiber supplement on the microbiome of the young rower at high-intensity exercising loads. Several consistent patterns in the gut microbiota were observed. First, the shifts induced by high exercise and dietary fibers were restricted to a limited number of phyla and genera, but were remarkable at the species level contributing to energy production. Second, the magnitude of change in microbial alpha diversity upon fiber consumption was drastic, constituting a 20.3% drop in diversity, by substantially enhancing the *Firmicutes*/*Bacteroidetes* ratio. Third, as suggested previously (41, 42) but now confirmed by this longitudinal case study, microbial response to dietary fiber consumption included the keystone species of the individual.

Our data showed that fiber consumption at high exercise loads led to decreased alpha diversity of the gut microbiota (Figure 2). Recent findings suggest a dynamic positive relationship between gut microbiota diversity and physical activity as professional athletes exhibit more diverse composition [ref (43)]. Paradoxically, in our study, there was a significant drop in alpha diversity upon dietary fiber consumption, most conceivably due to the rise in select advantageous bacterial species, such as those involved in butyrate production (Figure 5). Overall, these data support the view (44) that complex fibers of the dietary mix are highly selective for specific bacteria. It has become clear that animals can get by and often have high fitness with low-diversity microbiota (45, 46). Also, high gut microbial diversity has been linked to longer colonic transit time and systemic circulation of potentially harmful protein degradation products (47). Therefore, interpreting the health of the athlete's gut based on alpha diversity values of the microbiome implies a personal approach.

We observed a strong association between exercise loads, fiber intake, and *F*/*B* ratio (Figure 3), in good agreement with previous findings (48, 49). The proportional composition of the phyla clearly differentiated the subject from the matched control cohort with the abundance of *Firmicutes* and *Prevotella*. The *Prevotella* enterotype is supported by the diet of the subject in good support with earlier findings (50, 51). The dietary fiber intake has a limited influence on the communal stability of the latter two as compared with the baseline (Figure 3). However, dietary fiber intake resulted in enhanced abundance of *Prevotella* and *Roseburia* that became 41.7 and 4.2% more abundant, respectively, as compared with the time point of high competition (Figure 4). A recent *in vitro* study elucidating the mechanism of action of select dietary fibers on gut microbiota found that beta-glucan from oats induced the growth of *Prevotella* and *Roseburia* with a concomitant increase in SCFA propionate production (52). This study also showed that non-digestible sugars like inulin and oligosaccharides increase SCFA levels (52). Thus, our data allow concluding that non-digestible carbohydrates of the dietary fiber supplement promoted the growth of beneficial microorganisms for the performance of the athlete. In addition, there was less *Actinobacteria* (Figure 3), in harmony with previous studies (53). Interestingly, *P*/*A* was found to be <1 for all samples (0.5, 0.3, and 0.1), suggesting that decreased abundance of the *Proteobacteria* was linked to high exercise loads [see also (54)]. Of note, *Proteobacteria* are a major group behind the gut's metagenome functional variability (55).

Furthermore, we observed opposite dynamics of lactate- and acetate/butyrate-producing bacteria in periods of competition and upon dietary fiber intake, supporting the mechanism where during exercise the gut supplies lactate (56) and acetate (57) as fuel energy source (58). Though, generally fiber tends to increase SCFA-producing bacteria such as *Bacteroidetes* and *Actinobacteria* and decrease *Firmicutes* (59) as also observed in our study, whether it is a direct cause or because of changes in training and competition routine (outdoor vs. indoors rowing) along with dietary fiber intake that might have changed the metabolism needs further evaluation.

Our data show that high-endurance exercise and a prebiotic fiber-supplemented diet resulted in significant shifts across the key genera. We found that seven genera, namely *Prevotella, Parabacteroides* (Bacteroidetes), *Faecalibacterium, Ruminococcus, Coprococcus, Lachnospira* (Firmicutes), and *Corynebacterium* (Actinobacteria), were reduced upon high exercise loads, but the levels of these were restored upon dietary fiber consumption. These findings suggested that these seven genera affected primarily the levels of acetate and propionate available to the host. Both of these SCFAs are the known substrates for energy production, as well as in skeletal muscle (60, 61). In contrast, six genera, namely *Streptococcus* and *Dialister* (Firmicutes), *Bacteroides* (*Bacteroidetes*), *Bifidobacterium* (*Actinobacteria*), *Akkermansia* (*Verrucomicrobia*), and *Sutterella* (*Proteobacteria*), were specifically stimulated at high exercise loads, but inhibited by dietary fiber intake. These findings suggested higher butyrate production upon dietary fiber consumption with also potentially ameliorative effects on gut mucosal inflammation and oxidative status (62, 63). Elite athletes' dietary plans are based on the consumption of certain micronutrients, but the health of the gut microbiota is rarely considered (27). Here, we show that despite the individual features of the microbiota composition of the athlete, exercise-driven prevalence of acetate- and propionate-producing species was flexibly switched to butyrate producers after dietary fiber intake.

Baseline bacterial composition has repeatedly been observed to be a key factor of changes in the gut microbiota following dietary interventions (17, 36). What was noticeable upon dietary fiber consumption by the athlete was the increase in taxa, such as *F. prausnitzii*, with known beneficial effects on muscle function (15). However, in contrast to the published studies, we found that while having a positive effect on bacterial families associated with athletic excellence, fiber intake had detrimental contracting effects on the overall microbial community. This was surprising although in concordance with the notions that the host might lack keystone species (64) or lack strains to utilize specific dietary fiber (65). It is expected that the microbiome reverts to its original state after short-term dietary interventions (33, 34), although, positive impacts on the gut microbiota could be maintained for at least a year (12).

### Strengths

The major strength of the study was that it was conducted in “real-life” scenario as the temporal dynamics of the athlete's microbiota was explored by combining high-endurance exercise specifically with the athlete-designed dietary fiber supplement. The benefits of this study may lead to new insights into the cumulative effects a particular physiological interference has on the gut microbiome, that is on the role of the host's enterotype (with defined keystone species) has on the covariation of microbial communities upon dietary shifts and at high exercise loads. Finally, this is an individual athlete's study, and as such, it does not allow to draw solid and supportive conclusions. The value of the study lies in the aspect that overall variability in the physiological response of athletes to training and nutrition has not yet been adequately explored.

### Limitations

Firstly, it was not possible to fully control dietary intake, although, the participant was instructed to maintain normal habits. Secondly, the study did not examine the causal relationship between exercise performance and the gut microbiota of the athlete, although, a high number of intestinal bacteria of the *Veillonella* family that would be of advantage to the athlete were observed. Also, recording of metrics inflammation and immunosuppression could have helped to examine the microbial gut stress levels of the athlete. Finally, given that microbiome sequencing had a limited capacity to resolve taxa to the species level, therefore, the study focused on the proportionally largest reservoir of multiple species' diversity and functionality.

## Conclusions

Testing the microbiome of young athletes is necessary to obtain information on the dynamics and composition of this activity during the training process and at competition. At high levels of endurance exercise, athletes may have serious problems getting the amount of energy they need, so taking supplements to increase or to recover gut microbiota diversity at times of physical exertion is highly recommended. Our observations suggest that the dietary fiber-supplemented diet produces pronounced changes in the gut microbiota of the subject with high fractions of *Bacteriodetes* (*Prevotella*). This fact solely could be used to stratify athletes by their baseline gut bacterial composition before assigning such a fiber-supplemented diet. We evidenced that high dietary fiber intake at high exercise loads might produce profound changes beneficial to human health. Establishing the causal role of the GI microbiota and the underlying mechanisms would remain essential for the development of improved next-generation personal nutritional strategies. Only this type of in-depth understanding will allow for the selection of dietary fibers (or) mixtures thereof, to systematically target specific features of the GI microbiome (i.e., specific taxa, diversity, metabolites) with the goal of alleviating the immunometabolic features (frequently dysbiotic) that are characteristic of athletes' gut.

## Data Availability Statement

The raw data supporting the conclusions of this article will be made available by the authors, without undue reservation.

## Ethics Statement

Ethical review and approval was not required for the study on human participants in accordance with the local legislation and institutional requirements. The patients/participants provided their written informed consent to participate in this study.

## Author Contributions

MJ, UT, TT, and KP collected the samples, carried out the analysis, wrote the paper, and reviewed and accepted the final version of the manuscript. KP (corresponding) is responsible for the integrity of the work as a whole. All authors have read and agreed to the published version of the manuscript.

## Conflict of Interest

The authors declare that the research was conducted in the absence of any commercial or financial relationships that could be construed as a potential conflict of interest.
